# Substance use among second-generation immigrants in France: heritage language as a protective factor

**DOI:** 10.3389/ijph.2026.1609152

**Published:** 2026-06-15

**Authors:** Stéphane Legleye, Myriam Khlat, Damien Bricard

**Affiliations:** 1 INSERM U1018 Centre de Recherche en Épidémiologie et Santé des Populations (CESP), Villejuif, France; 2 Ecole nationale de la statistique et de l’analyse de l’information (ENSAI), Bruz, France; 3 Institut National D’études Démographiques (INED), Paris, France; 4 Montpellier recherche en économie (MRE), Université de Montpellier, Montpellier, France; 5 Institut de recherche et de documentation en économie de la santé (IRDES), Paris, France

**Keywords:** France, heritage language, parentage, second-generation immigrants, substance use

## Abstract

**Objectives:**

Study substance use among second-generation immigrants in France (G2) in comparison with first-generation immigrants (G1) and majority population, and particularly the variation according to practice of heritage language.

**Methods:**

in a nation-wide probability sample, we focused on the two largest groups (from Maghreb and Southern-Europe: n = 2,736), analysing their use of alcohol, tobacco and cannabis. The main factor of interest was practice of heritage language at home at age 15 (vs. exclusive use of French), and we also considered type of parentage: homogenous (two immigrant parents from the same country) vs. mixed (only one, the other being French).

**Results:**

In comparison with the majority population (neither G1 nor G2: n = 19,185), G1 had lower levels of substance use, followed by G2 speaking the heritage language and by G2 with homogeneous parentage. The other G2 reported higher levels of substance use, close to those of the majority population (even higher for tobacco among Maghrebins and cannabis among South-Europeans). These associations remained robust after adjusting for potential individual and contextual confounding variables.

**Conclusion:**

Integrating those specificities may help in designing culturally grounded prevention policies benefitting the population at large.

## Introduction

Adolescents and adults of immigrant origin constitute a significant and growing proportion of the population in many countries of the West, and particularly in Europe [1]. In this context, their experiences and life outcomes have become a focus of scholarly research, despite inherent methodological challenges [2].

Of particular interest is the degree to which this so-called “second generation” engages in the society at large, while also honouring their cultural heritage [3]. It has been argued that scholars have focused on the adoption of host society culture, overlooking to some extent the importance of retaining the heritage culture [4]. Many studies of the relationships between acculturation, health and health behaviours have indeed considered acculturation as essentially a process of assimilation [5]. And yet, a recent literature review has concluded that, among immigrant youth in the United States, there were more protective than risk-enhancing cultural values against alcohol and other drug use, calling for further research in other cultural contexts [5, 6].

In the European Union, France is a favourable context for such study as it has one of the largest populations of second-generation immigrants. In 2019, approximately 12% of the French population had one or both parents born abroad (with an additional 10% having at least one grandparent born abroad) [7]. Second-generation immigrants of non-EU origin in France encounter difficulties in social integration and experience significant hardships in educational attainment [8], employment [9] and income [10–14]. Generally speaking, poorer socio-economic status and living conditions associated with stressful experiences related to racial discrimination in education and employment can increase the risk of substance abuse among minority populations, and especially the second generation [15]. Traditional values held by immigrant communities may help mitigate this risk, as generally the substance use levels of the first-generation immigrants are lower than those of their native counterparts [16–18].

Cultural transmission is a complex process, involving different domains of acculturation (involvement with host country culture) and of so-called “enculturation” (involvement with family of origin norms and values) [3]. Having two foreign-born parents, rather than only one, is a traditional measure of acculturation widely used in epidemiological and social research [19–23]. It primarily captures the structural conditions for family cultural transmission, rather than the actual extent or success of such transmission. When possible, focusing on effective transmitted cultural behaviours seems more relevant. In research, language spoken at home is also frequently used as a proxy for acculturation [24], but typically with more focus on the acquisition of the host country’s language rather than on the transmission of the parents’ native language [25–29]. However, the acculturation framework cannot be applied as such for G2, as they grow up in the country and acquire the host country language at school: it seems more relevant to turn around the perspective, with the practice of the heritage language as a proxy of cultural transmission. Immigrant youth who hear at home their ‘heritage language’ (language of parental country of origin) reap benefits in terms of cultural and ethnic identity development [30]. It has also been suggested by education specialists that preserving heritage languages while acquiring the majority language has protective value for children’s academic outcomes, cognitive development, and social-emotional wellbeing [31].

A systematic literature review on substance use among adolescents in Europe provided evidence for a healthier behaviour profile among immigrants than among native-born adolescents regarding alcohol use [32], a difference which may be related to the influence of heritage values and practices among immigrants. The extent to which heritage language practice at home influences substance use behaviours among immigrants’ offspring has not been studied.

This study focuses on substance use in France among second generation immigrants (G2), in comparison with first-generation immigrants (G1) and the majority population (neither G1 nor G2). For second generations, the relationship with the practice of heritage language was examined, also considering type of parentage (two immigrant parents from the same country vs. only one, the other being French). The main research questions were the following: (1) how do the substance use of second-generation immigrants compare to those of first-generation immigrants and to those of the population with no immigrant background, and; (2) among second generations, how does substance use vary depending on the practice of the parental language of origin?

We used survey data from France, focusing on consumption of alcohol, tobacco and cannabis for the two largest immigrant groups in the country, originating respectively from Southern Europe and North Africa [33].

As alcohol and tobacco consumption are linked to gender, in the general population as well as in the first-generation immigrant population [34–36], we tested for the interactions between gender and spoken language or type of parentage. Finally, we also tested for the interaction between heritage language practice and type of parentage.

## Methods

### Data

The Health Barometer is a series of cross-sectional probabilistic surveys conducted in France since 1992 [37]. The 2017 “Health Barometer” is a nationwide French telephone survey that used a two-stage random (household/individual) sample and a dual sampling frame (landline/mobile line) to measure health perceptions and behaviours of the general population. This investigation was approved by the French Commission on Individual Data Protection and Public Liberties (Commission Nationale Informatique et Libertés (CNIL)), and all data collected were anonymous and self-reported. The response rate, calculated using the AAPOR formula 2 [38], was 48.5%. The number of telephone numbers (landlines and mobile) available to join the selected person within the household was used to compute pseudosampling weights; final weights were obtained through a calibration procedure considering cross-classification of gender and age (10 categories), number of household members, educational level, size of the urban area and region of residence, in order to match the distribution of the last national Labor Force Survey. The detailed methodology can be found in [39].

The initial sample of respondents comprised 25,319 individuals aged 18–75 years who answered questions about their geographical origin, health behaviours and self-reported health. Results on first generation immigrants have been published using previous editions of the Health Barometer [35, 36] or the present one [34]. However, none of them specifically studied second generations or considered intrafamilial cultural transmission.

### Outcomes

The outcome variables were based on standard indicators for monitoring drug use. Daily tobacco smoking was defined as the reporting of smoking at least one tobacco cigarette per day (yes/no). Regular alcohol drinking was defined by the report of, in the last 30 days, at least 2 days of consumption per week of any alcoholic beverage among the four following types: wine, beer, spirits and other types of beverages (cider, champagne, porto wine etc.). Cannabis use was defined as having smoked cannabis at least once in the last 12 months.

### Variables of interest

Immigrant status was categorized using six variables: the individual’s country of birth and nationality at birth, as well as the country of birth and nationality of his/her parents. We selected two emigration regions of interest, based on the share of respondents in the national population [7]: North Africa (Tunisia, Morocco and Algeria) and South-Europe (Italy, Spain, Greece and Portugal); other regions were grouped into an “Other” category. The respondents who emigrated from these regions were considered G1 (first generation immigrant). Those who had at least one parent who had been citizen of a country in these regions were called G2 (second generation immigrant). G1 and G2 from North Africa were referred to as “Maghrebins”. In the French context, this term refers to individuals from the North African region and their descendants (excluding those of European or Jewish origin). First and second generations of immigrants from South Europe were referred to as “South-Europeans”. The 10 respondents who had a parent from Maghreb and another from South-Europe were considered Maghrebins.

Among G2, we distinguished between homogenous parentage (both parents from the same region) and mixed parentage (parents from different regions). In the latter case we consider the following categories of interest: a parent from North Africa and another parent born elsewhere (including France); a parent from South Europe and a parent born elsewhere (including France).

For the respondents with at least one parent born abroad (i.e., G1 and G2), one question described the practice of the parental foreign language (heritage language) by the respondents: “At home, when you were 15, did you occasionally or fluently speak a language other than French with your parents? Yes/no [Please cite]”. The heritage languages were recoded manually and categorized into two groups for this study [1]: Arabic, Kabyle and Berber, and [2] Italian, Spanish, Portuguese and Greek, corresponding to the two selected regions of origin. In case where languages from both regions were spoken, priority was given to the Maghreb category.

Finally, the first variable of interest distinguishes G1 and G2 within the two regions of interest. The second variable concerns only G2 and their use of their heritage language at age 15: those who spoke it occasionally or fluently (in addition to French, if applicable) are hereafter named ‘HL-speakers’ (heritage language speakers) and those who do not and used only French are named ‘non-HL-speakers’. The type of parentage (homogenous vs. mixed) of the respondents was considered, as it is related to cultural transmission within the family.

### Other independent variables

Several control variables were introduced to account for compositional differences across immigrant generations, origin groups, and language spoken at home. We considered age, sex, living in couple (yes/no), living with at least one child (yes/no), household size (five categories: 1-5+), perceived financial situation of the household (3 categories: at ease, just enough, it is hard), the tercile of standard of living of the household, and the deprivation index of the city of residence (in quintiles) [39] and whether the respondent lived in the Paris metropole area, which hosts the largest share of immigrants in France. Educational attainment was categorized into five levels based on ISCED classification [40], ranging from below upper secondary education to Bachelor’s degree or equivalent. To compare populations of different ages and education levels, while accounting for historical changes in educational attainment [41, 42] that differed for both sexes, we computed *ridit* scores for the educational level [43]. These scores represent, for each sex, a ranked variable considering three age groups (18‐39), (40‐59), (60‐75). We favoured this approach over simply adjusting for educational level, to better disentangle the effects of education from that of cultural transmission and to provide a clearer estimate of the moderating effect of sex in this process.

### Missing values

There was no missing value for the variables of interest, age, sex and educational level; the missing value rates were very low for all the other variables (less than 1%), except for the household standard of living, whose missing values were treated as a separate category.

### Analytical sample

The analytical sample is restricted to 22,551 respondents, and is composed of G1 originating from the Maghreb or Southern Europe, G2 with at least one parent originating from these regions, as well as individuals whose parents were both born in France (the reference group). The 2,768 excluded respondents originated from countries that were either too diverse or insufficiently represented to allow for meaningful analyses.

### Statistical analysis

We used bivariate analyses with Chi-square tests to describe the distributions of the three outcomes in the sample. Multivariable Poisson regression models with robust variance [45] were used to estimate risk ratios (RR) for the three outcomes. Those models were run for our variables of interest, adjusting for all control variables. Previous studies have reported significant gender differences in substance use among first-generation immigrants, particularly those from non-Western countries [34–36]. To assess whether similar patterns persist in G2 according to family cultural transmission, we conducted interaction tests. In addition, the interaction between language spoken and type of parentage was examined.

Individual and contextual variables may play a role as confounders when analysing associations of living conditions and socioeconomic status with the study outcomes. However, some of these control variables may be inter-related, leading to over-adjustment bias, or may intervene within the causal pathway leading from exposure to outcome. This is particularly true for perceived financial situation of the household, deprivation index of the city of residence, and educational level. To address this question, we conducted sensitivity analyses comparing risk ratios across four model specifications: Fully adjusted models (Tables 3, 4), Models adjusted only for sex and age (Supplementary Tables 3-1, 4-1), Models adjusted for all covariates except the three potential mediators (Supplementary Tables 3-2, 4-2), Unadjusted models (Table 2). Results were consistent across specifications, indicating robustness to model choice.

Finally, we replicated the multivariable models using parentage as a substitute for language spoken at home (Supplementary Table 5, 5-1, 5-2 for the models with full adjustments, and with the same two limited sets of variables as above, respectively). Since the results regarding language spoken and parentage yielded consistent estimates, only the primary analyses were presented in the main text.

Confidence intervals and p-values (present in the tables) are not reported in the text unless they are specifically interpreted. All statistical analyses were performed using SAS V9.4; final weights were considered in all analyses.

## Results

### Descriptive statistics

The analytical sample included 898 first generation (G1) immigrants (380 from South Europe and 518 from Maghreb), 1,838 s generation (G2) immigrants (1,183 from South Europe and 655 from Maghreb) and the reference group comprising 19,185 individuals born French in France with no immigrant parent. Among G1, the proportion of mixed parental couples were 4.5% in South-Europeans and 14.4% in immigrant from Maghreb. Among G2, the proportions were 65.7 among South-Europeans and 39.5% among Maghrebins (Table 1). In G2 with homogenous parentage, 96% of the parents originate from the same country among South-Europeans, 97% among Maghrebins. In G2 with mixed parentage, a large majority had a parent born French (95% among G2 from South-Europe, 88% among G2 from Maghreb).

**TABLE 1:** Foreign language spoken with parents according to immigrant groups (Continental France, Health Barometer, 2017).

​	In sample		Homogenous parentage		Mixed parentage		​
​	N	%	N	%	N	%	Total (%)
G1 Southern Europe	380	—	354	95.5	26	4.5	100
G1 Maghreb	518	—	421	85.6	97	14.4	100
G2 Southern Europe	1,183	—	426	34.3	757	65.7	100
HL-speaker	463	39.3	288	57.7	175	42.3	100
Non-HL-speaker	720	61.7	138	19.2	582	80.8	100
​	​	100.0	​	​	​	​	​
G2 Maghreb	655	—	399	60.5	256	39.5	100
HL-speaker	356	53.5	286	77.9	70	22.1	100
Non-HL-speaker	299	46.5	113	40.4	186	59.6	100
​	​	100.0	​	​	​	​	​

G1 and G2: first and second generations of immigrants.

HL‐speaker / non‐HL‐speaker: speaker of the Heritage Language at home / non-speaker of the HL i.e. exclusive speaker of French, at age 15.

Among G2, speaking the heritage language at age 15 (HL-speakers) was more common among Maghrebins (53.5%) than South-Europeans (39.3%), and over twice as common in individuals with two foreign-born parents. This may be explained by the fact that in mixed parentage couples, one parent is often French, which may influence language transmission within the household. G1 from Southern Europe were older, whereas those from Maghreb were younger than the reference group (Supplementary Table 1). Among G1, Maghrebins had the highest educational level, which was comparable to that of the majority population; they were also the poorest and lived more frequently in larger households with children. Among G2, Maghrebins were the youngest and were poorer, living more often in larger households with children, and in the Paris region.

For G2, the two criteria of cultural transmission are correlated: the proportion of homogenous parentage among HL-speakers was higher than among French-only speakers (non-HL-speakers). For those from the Maghreb, the figures were 77.9% vs. 40.4% and for those from Southern Europe, 57.7% vs. 19.2% (Table 1).

In the reference group, levels of regular alcohol use, tobacco smoking and past-year cannabis use were 26.0%, 27.0% and 9.3%, respectively (Table 2). G1 Maghrebins had the lowest levels of regular alcohol and past-year cannabis use (6.7% and 3.9%), while South-Europeans showed intermediate levels between Maghrebins and the reference group. Compared to G1, G2 Maghrebins and South-Europeans had higher tobacco and past-year cannabis use. Alcohol use remained lower for G2 Maghrebins, but it was unchanged for South-Europeans compared to G1. Language use was unrelated to substance use among G2 South-Europeans, but among G2 Maghrebins, HL-speakers had lower alcohol and tobacco use (Table 2).

**TABLE 2:** Substance use levels according to immigrant groups (Continental France, Health Barometer, 2017).

​	N	Alcohol		​	Smoking		​	Cannabis		​
​	​	%	RR^a^	RR^b^	%	RR^a^	RR^b^	%	RR^a^	RR^b^
Majority population	19,815	26.0	Ref.	​	27.0	Ref.	​	9.3	Ref.	​
G1	898	**12.3**	0.47	​	**25.9**	0.96	​	**5.2**	0.56	​
Southern Europe^a^	380	**21.3**	0.82	​	23.3	0.86	​	**4.6**	0.49	​
Maghreb^a^	518	**6.7**	0.26	​	27.6	1.02	​	**3.9**	0.42	​
G2 Southern Europe	1,183	**19.9**	0.77	​	**31.5**	1.17	​	**11.6**	1.25	​
HL-speaker	463	**18.5**	0.71	Ref.	**32.4**	1.20	Ref.	10.0	1.08	Ref.
Non-HL-speaker^b^	720	**20.8**	0.80	1.12	**31.0**	1.15	0.96	**12.7**	1.37	1.27
G2 Maghreb^a^	655	**11.4**	0.44	​	**37.6**	1.39	​	**15.1**	1.62	​
HL-speaker	356	**4.6**	0.18	Ref.	**30.1**	1.11	Ref.	**12.3**	1.32	Ref.
Non-HL-speaker^b^	299	**19.2**	0.74	4.17	**46.2**	1.71	1.53	**18.3**	1.97	1.49

Majority population = neither G1 nor G2.

G1 and G2: first and second generations of immigrants.

HL‐speaker / non‐HL‐speaker: speaker of the Heritage Language at home / non‐speaker of the HL i.e. exclusive speaker of French, at age 15.

In bold: Chi2 test p-value <0.05 when comparing the category to the majority population; Underlined: Chi2 test p‐value <0.1.

RR: risk ratio; Ref. = reference for the risk ratio RR.

^a^ Risk ratio of the category population compared to the majority population.

^b^ Risk ratio of the non-HL-speakers compared to the HL-speakers of the same origin.

### Multivariable analysis

#### Substance use by immigration generation, origin and language

Compared with the majority population (Table 3, upper panel), G1 from Southern Europe had similar consumption levels for all three substances, but G2 showed lower level of alcohol consumption (RR = 0.80) and slightly higher levels of smoking (RR = 1.10, p = 0.078) and cannabis (RR = 1.30). South-Europeans G2 drank as much as G1 (RR = 0.91, p = 0.434), but had higher levels of tobacco smoking (RR = 1.23, p = 0.064) and cannabis (RR = 1.67) (Table 3, Contrasts).

**TABLE 3:** Multivariable modelling of substance use according to origin (Continental France, Health Barometer, 2017).

​	Alcohol				Smoking				Cannabis			
​	RR	LCL	UCL	P	RR	LCL	UCL	P	RR	LCL	UCL	P
Comparison to the majority population												
G1 South Europe	0.87	0.71	1.07	0.201	0.89	0.73	1.08	0.253	0.78	0.49	1.17	0.259
G1 Maghreb	**0.29**	0.21	0.38	<0.001	**0.81**	0.70	0.94	0.005	**0.33**	0.22	0.47	<0.001
G2 South Europe	**0.79**	0.69	0.90	<0.001	1.10	0.99	1.22	0.086	**1.30**	1.09	1.54	0.003
G2 Maghreb	**0.48**	0.39	0.59	<0.001	1.02	0.91	1.14	0.736	0.94	0.78	1.12	0.517
Contrasts												
South-Europe: G2 vs. G1	0.91	0.71	1.16	0.435	1.23	0.99	1.53	0.064	**1.67**	1.05	2.66	0.030
Maghreb: G2 vs. G1	**1.69**	1.20	2.39	0.003	**1.26**	1.05	1.50	0.012	**2.83**	1.88	4.26	<0.001
G2: South Europe vs. Maghreb	**1.64**	1.30	2.08	<0.001	1.08	0.93	1.25	0.343	**1.38**	1.08	1.76	0.009

Majority population = neither G1 nor G2.

G1 and G2: first and second generations of immigrants.

Adjustments for: generation and region of origin + age (linear and quadratic terms), sex, educational level (ridit score), household characteristics (living in a couple, household size), socioeconomic status (perceived financial situation, standard of living, deprivation index of the city of residence), and geographic location (living in the Paris metropolitan area).

RR, risk-ratio, LCL-UCL: lower and upper 95% confidence interval limits.

Reference = majority population (neither G1 nor G2), except in the “Contrasts” panel.

Bold and underlined type: p-value<0.05 and <0.1 respectively.

Of all groups, Maghrebin G1 had the lowest levels of substance use relative to the majority population (i.e., the reference group): RR = 0.29 for alcohol, RR = 0.83 for smoking and RR = 0.34 for cannabis (Table 3, upper panel). G2 from the Maghreb had lower levels of alcohol (RR = 0.49), but their levels of smoking and cannabis were not significantly different from those of the reference group. Comparison between G1 and G2 (Table 3, Contrasts) showed that G2 had higher levels of substance use than G1 (RR = 1.70 for alcohol, RR = 1.26 for smoking and RR = 2.80 for cannabis).

G2 from Southern Europe had higher levels of alcohol (RR = 1.64) and cannabis use (RR = 1.38) than those from the Maghreb (Table 3, Contrasts). However, their levels of tobacco smoking were comparable.

Looking at variation of substance use according to heritage language practice within G2 (Table 4, Contrasts) we found no significant differences in South-Europeans, while non-HL-speakers had higher levels of alcohol and tobacco consumption than HL-speakers among Maghrebins: RR = 3.85 for alcohol, RR = 1.47 for smoking, and a similar but non-significant estimate was observed for cannabis (RR = 1.37, p = 0.079). Therefore, compared to the majority population, HL-speakers in G2 (especially Maghrebins) tended to have lower levels of substance use, whereas non-HFL-speakers tended to have more similar levels of use (levels were even higher, for alcohol among Maghrebins and cannabis among South-Europeans (Table 4, upper panel)).

**TABLE 4:** Multivariable modelling of substance use according to language practice (Continental France, Health Barometer, 2017).

​	Alcohol				Smoking				Cannabis			
​	RR	LCL	UCL	P	RR	LCL	UCL	P	RR	LCL	UCL	P
Comparison to the majority population												
G2 South Europe												
HL-speaker	**0.74**	0.60	0.92	0.007	1.07	0.91	1.26	0.399	1.10	0.81	1.45	0.532
Non-HL-speaker	**0.83**	0.70	0.97	0.022	1.11	0.97	1.27	0.113	**1.44**	1.16	1.77	0.000
G2 Maghreb												
HL-speaker	**0.20**	0.13	0.30	0.000	**0.83**	0.70	0.98	0.036	0.80	0.60	1.03	0.100
Non-HL-speaker	**0.78**	0.61	0.97	0.030	**1.23**	1.05	1.42	0.007	1.09	0.85	1.37	0.471
Contrasts												
G2 vs. G1 (ref.)												
South-Europe												
HL-speaker	0.85	0.63	1.14	0.282	1.20	0.93	1.55	0.152	1.41	0.84	2.37	0.194
Non-HL-speaker	0.94	0.73	1.22	0.665	1.25	0.99	1.58	0.064	**1.85**	1.14	2.99	0.012
Maghreb												
HL-speaker	0.71	0.43	1.19	0.196	1.02	0.82	1.28	0.827	**2.40**	1.52	3.78	0.000
Non-HL-speaker	**2.72**	1.89	3.90	0.000	**1.51**	1.23	1.85	0.000	**3.28**	2.12	5.07	0.000
G2: Non-HL-speaker vs. HL-speaker (ref.)												
G2 South-Europe	1.11	0.85	1.45	0.443	1.04	0.84	1.28	0.724	1.32	0.92	1.89	0.136
G2 Maghreb	**3.85**	2.33	6.25	0.000	**1.47**	1.18	1.85	0.000	1.37	0.96	1.96	0.079

Majority population = neither G1 nor G2.

G1 and G2: first and second generations of immigrants.

Adjustment on generation, region of origin and language (“speakers” and “non-speakers”) + age (linear and quadratic terms), sex, educational level (ridit score), household characteristics (living in a couple, household size), socioeconomic status (perceived financial situation, standard of living, deprivation index of the city of residence), and geographic location (living in the Paris metropolitan area).

HL-speaker / non-HL-speaker: speaker of the Heritage Language at home / non-speaker of the HL i.e. exclusive speaker of French, at age 15.

RR risk-ratio, LCL-UCL: lower and upper 95% confidence interval limits.

Reference = majority population (neither G1 nor G2), except in the “Contrasts” panel.

Bold and underlined type: p-value<0.05 and <0.1 respectively.

Consequently, among G2, non-HL-speakers had higher levels of substance use than G1 of the same origin, especially for Maghrebins (Table 4, Contrasts). We found similar results when distinguishing G2 by parentage: those with mixed parentage had higher substance use than those with two foreign-born parents (Supplementary Table 5).

#### Sensitivity checks

Estimates from the model adjusted only on sex and age or with the limited set of control variables were very similar to those from the fully adjusted model, suggesting that the effects of immigrant generation, region of origin and language are prominent and not confounded by family and contextual factors (Supplementary Tables 3-1, 3-2). The same was true for HL-speaking among G2 (see the comparison between Table 4; Supplementary Tables 4-1, 4-2). In addition, the adjusted risk-ratios in Table 4 were very close to the unadjusted ones in Table 2. For example, the unadjusted RR comparing the Maghrebin HL-speakers to the reference group for alcohol is 0.18 (Table 2) vs. 0.20 for the fully-adjusted one (Table 4). The same is true for the contrasts comparing non-HL-speakers to HL-speakers among G2: for example, the unadjusted RR comparing the non-HL-speakers to the HL-speakers in Maghrebins is 4.17 (Table 2) vs. 3.85 in the fully adjusted model (Table 4).

#### Interactions

To assess potential effect modification, we tested two-way interactions between sex and region of origin, sex and parentage and sex and language spoken at home on substance use outcomes among G2. No significant interactions were observed for alcohol (p = 0.496), smoking (p = 0.934), or cannabis use (p = 0.361) by region of origin. Similarly, neither language spoken at home nor type of parentage interacted significantly with gender for any outcome (all p > 0.3).

In the South-European G2 subgroup, no interactive effects of type of parentage and language spoken at home were detected (all p > 0.360). Among G2 individuals of Maghreb, a borderline interaction was found for alcohol use (p = 0.053), with the highest risk observed in those who did not speak a foreign language at home and had mixed parentage; no such interactions were noted for smoking or cannabis (all p > 0.2).

## Discussion

### Summary of the results

Looking at the first-generation immigrants, in comparison to the reference group (neither G1 nor G2), we find that those from the Maghreb have a more favourable profile of substance use than those from Southern Europe. The second generations do not have a similarly advantageous profile, except for alcohol consumption for those originating from the Maghreb. Notably, compared to the reference group, second generations originating from Southern Europe have a significantly greater use of cannabis while those from Maghreb have higher use of tobacco. In addition, among G2, those who only spoke French at home or those who had a mixed parentage showed higher levels of substance use than the others and sometimes higher levels than the majority population. Globally, lower levels of cultural transmission were associated with higher levels of those unhealthy behaviours. We found that both indicators of family cultural transmission are correlated and yield similar results. In addition, we found that all results remain largely unchanged when adjusting for various series of confounding factors (including no control at all). Although the potential for unmeasured confounding, mediators or residual over-adjustment cannot be entirely ruled out due to the uncertainty surrounding the true causal structure, we may therefore safely conclude that practices related to language spoken at home or parental origin play an independent role in shaping the substance use levels of G2 in France. Nonetheless, measuring language practice presents a practical and methodological advantage: it requires only one question, simplifying data collection and reducing reliance on respondents’ recall accuracy. In contrast, assessing parental nativity involves four questions about the migratory history of both parents—questions that may be perceived as sensitive in certain contexts.

### Interpretations and limits

The differences which are observed, with less of an advantage in the second generations, may be interpreted within the framework of the “healthy migrant effect” extended to substance use (known as “immigrant paradox in substance use” [46]). Second, according to the “cultural effects hypothesis [47], immigrants are influenced in terms of their lifestyle by the cultural norms of their country of origin. Depending on the way the immigrants raise their children, those norms may be transmitted to the second generations, and if they are, then those children may benefit as well from a transmission of favourable health outcomes [48]. In one study conducted in the US, the observation of a “substance use paradox” was interpreted in relation with the protective role of the family, and it was posited that conversely acculturation would be accompanied by a learning and preferred practice of English in non-English proficient families, with a weakening of family ties [46].

The two sets of findings for respectively the first and second generations accord with both the theories of “immigrant paradox in substance use” and “cultural effects hypothesis”. The G1 from the Maghreb have much more favourable health behaviours than the reference group. This type of restraint reflects the influence of Islam, which, of all religions is the one which takes the clearest stand against unhealthy behaviour, and particularly the consumption of alcohol. In a cross-national study of immigrants’ health in Europe, it was shown that the decisive factor for healthier practices among immigrants was not religiosity, but rather the predominance of Islam among the different religions in the country of origin [49], and that the healthy lifestyles were found also among the offspring.

The difference between the two groups of first-generation immigrants is also explainable in terms of cultural factors and health transition stage: the countries from Southern Europe considered in this study (Italy, Spain and Portugal) are Mediterranean countries which are culturally close to France as well as close in terms of health infrastructure and mortality and morbidity profiles. Therefore, the differences between the first-generation immigrants from those countries and the majority population are expectedly limited, whereas the differences with the immigrants from the Maghreb, encompassing less advanced countries, lagging behind in the smoking epidemic process [36] and with more traditional dietary and health behaviours are large.

Comparing the substance use levels within G2 according to the practice of heritage language, we find no significant difference for the group from Southern Europe. In G2 from Maghreb, alcohol use and tobacco use levels were significantly higher in non-PFL-speakers (i.e., French only-speakers) than in PFL-speakers. In addition, alcohol consumption level remained well below the level of the reference group, whereas smoking level exceeded it.

This pattern of association between practice of heritage language and substance use in G2 from Maghreb in France accords with findings from a review of studies, mostly from the United States, highlighting how most values and cultural practices served as protective factors against alcohol and other drug use by immigrant youth [6]. Language use may be viewed both as one component of acculturation (when referring to the host country’s language) and as one component of enculturation (when referring to heritage language). As such, it has been considered part of a behavioural domain, along with food preferences, customs and social interactions, which may influence substance use among immigrant use [24]. Those conceptualizations were developed in relation with the acculturation profiles of first-generations immigrants, which have to learn the host country’s language upon arrival.

The situation is different for second generations born and schooled in the host country. In their case, the balance in their linguistic practices between heritage language and host country language is reversed compared to first-generation immigrants. Nevertheless, well integrated immigrants and their descendants (i.e., those with mixed parentage or who are non-HL-speakers as opposed to the others) may perceive more easily discriminations than the others because they are more in competition with successful natives in key social domains (e.g., employment, housing), while they are more exposed to public and local news and are more conscious of the discrepancies between public discourses and their personal experiences [50]. This may explain why the most acculturated second generations tend to present higher levels of some substance use than the reference group (here tobacco for Maghrebins and cannabis for Southern-Europeans). In particular, that may explain why acculturation or enculturation is associated with stronger differences in substance use in G2 Maghrebins than in G2 South-Europeans, because, as non-UE descendants, they experience more discriminations than the latter.

That our results are not confounded by a large series of sociodemographic characteristics confirms that family culture (parentage as well as heritage language spoken at home) is a primary determinant of identity and of health behaviours and should be considered for targeted prevention policies.

Our study has several limitations. The response rate of 48.5% is relatively high, but it does not rule out the possibility of selection bias among respondents, even if the use of weighting and statistical adjustments helps mitigate its impact. Another issue is the size of the G2 subsamples, which limits statistical power of the analyses; in particular, the absence of statistically significant interaction effects should therefore be interpreted with appropriate caution. A third limitation is that the age range of our sample (18–75 years) excludes two key groups: adolescents, who live through a critical period for the formation of health behaviours, and the older adults, whose experiences may differ substantially from those of younger cohorts.

There was no data on religion in the survey questionnaire, a major cultural factor likely to influence alcohol consumption patterns [32]. The type of parentage or the use of a heritage language may both facilitate the transmission of religious values and serve as a marker of such transmission. However, speaking a language should not be conflated with practicing a religion. Furthermore, factors beyond religious practice (cultural norms, health considerations, or social expectations) may also influence alcohol-related behaviours.

In France, linguistic transmission to children depends on the social background of immigrant parents [51]. However, we considered education and socioeconomic status only as confounders instead of categories that determine the health behaviours [52–54] or that could interact with heritage language or parentage in G2 [50]. A replication of this study using a larger sample may help providing a more comprehensive interpretation of mechanism at play.

As a conclusion, substance use among second generation immigrants is correlated with their heritage language practice, even after adjusting for socio-demographic characteristics. Particularly among Maghrebins, those who did not speak the heritage language consumed more substances than those who spoke it, and this got them closer to the majority population. Practice of heritage language is a very concrete measure of cultural transmission which is easy to collect with a simple question. It also reflects a competence that is valuable on cultural and scholarly grounds, and that can be considered less sensitive to collect in certain contexts than cultural traits or details of the migration history of the parents. Prevention policies should consider the cultural background and family heritage of second-generation youth, supporting the intergenerational transmission of health-promoting norms and behaviours.
