# Supplementary material for: Substance use among second-generation immigrants in France: heritage language as a protective factor
Source: Int J Public Health. 2026 Jun 15;71:1609152. doi: 10.3389/ijph.2026.1609152 (PMC13310806; doi:10.3389/ijph.2026.1609152)
Supplement: Supplementary file 1 [file Supplementaryfile1.pdf]

Online supplementary tables:

Supplementary Table 1: Descriptive statistics: demographic and socioeconomic characteristics by immigrant origin, generation and language practice

		Reference group	Southern Europe				Maghreb			
			G1	G2	G2 HL-speakers	G2 Non-HL-speakers	G1	G2	G2 HL-speakers	G2 Non-HL-speakers
	N	19815	380	1183	463	720	518	655	356	299
Age	Median	47,0	54,0	47,0	45,0	50,0	42,0	35,0	34,0	37,0
Sex	Women (%)	51,3	51,3	51,7	51,7	51,8	50,2	50,2	55,3	44,5
Education	> Bac (%)	33,8	17,8	28,3	28,7	28,1	28,9	27,5	31,4	23,0
Couple	Yes (%)	65,6	72,4	63,8	63,7	64,0	63,2	45,2	48,8	41,0
Children	Yes (%)	27,9	23,3	28,3	34,3	24,5	43,5	39,8	46,1	32,7
Employed	Yes (%)	58,2	54,5	57,8	34,5	47,3	48,9	58,1	40,8	43,1
	At ease (%)	63,1	61,9	61,4	64,8	59,2	44,0	50,4	46,4	55,1
Financial well-being	Difficult (%)	23,4	23,3	22,1	19,7	23,8	23,4	29,1	34,2	23,1
	Hard (%)	13,5	14,8	16,5	15,6	17,0	32,6	20,5	19,4	21,9
Household size (1-5+)	Mean	2,7	2,5	2,6	2,8	2,6	3,1	3,3	3,4	3,1
Poverty index (1-5)	Mean	3,0	2,9	2,9	2,8	3,0	2,8	3,0	3,1	2,9
Paris region	(%)	14,2	28,7	16,9	24,8	11,7	37,7	28,6	28,6	28,5

G1 and G2: first and second generations of immigrants.

HL-speaker / non-HL-speaker: speaker of the Heritage Language at home / non-speaker of the HL i.e. exclusive speaker of French, at age 15

Supplementary Table 3-1: Multivariable modelling of substance use according to origin adjusting for sex and age

	Alcohol				Smoking				Cannabis			
**Model 1**	**RR**	**LCL**	**UCL**	**P**	**RR**	**LCL**	**UCL**	**P**	**RR**	**LCL**	**UCL**	**P**
Comparison to the majority population												
G1 South Europe	**0.81**	0.66	0.99	0.049	0.97	0.79	1.17	0.736	0.81	0.51	1.21	0.339
G1 Maghreb	**0.26**	0.19	0.34	<.001	0.92	0.79	1.05	0.223	**0.38**	0.26	0.55	<.001
G2 South Europe	**0.77**	0.67	0.87	<.001	**1.16**	1.04	1.29	0.006	**1.32**	1.11	1.57	0.002
G2 Maghreb	**0.44**	0.36	0.53	<.001	**1.12**	1.00	1.26	0.045	0.99	0.83	1.18	0.955
Contrasts												
South Europe:												
G2 vs G1 (ref.)	0.94	0.74	1.20	0.626	1.20	0.96	1.49	0.103	**1.64**	1.03	2.60	0.037
Maghreb:												
G2 vs G1 (ref.)	**1.71**	1.21	2.42	0.002	**1.23**	1.03	1.47	0.024	**2.58**	1.72	3.89	<.001
G2: South Europe												
vs Maghreb (ref.)	**1.75**	1.38	2.22	<.001	1.03	0.89	1.20	0.681	**1.33**	1.05	1.69	0.020

Majority population= neither G1 nor G2.
G1 and G2: first and second generations of immigrants
Adjustment on generation (G1, G2), region of origin + sex and age (linear and quadratic terms).
RR risk-ratio, LCL-UCL: lower and upper 95% confidence interval limits).
Reference=majority population, except in the "Contrasts" panel.
Bold and underlined type: p-value<0.05 and <0.1 respectively.

Supplementary Table 3-2: Multivariable modelling of substance use according to origin adjusting for sex, age, household characteristics, standard of living and geographic location

	Alcohol				Smoking				Cannabis			
	**RR**	**LCL**	**UCL**	**P**	**RR**	**LCL**	**UCL**	**P**	**RR**	**LCL**	**UCL**	**P**
Comparison to the majority population												
G1 South Europe	**0.80**	0.65	0.98	0.034	1.01	0.82	1.22	0.934	0.79	0.49	1.19	0.289
G1 Maghreb	**0.26**	0.19	0.35	<.001	0.92	0.8	1.06	0.281	**0.35**	0.24	0.51	<.001
G2 South Europe	**0.77**	0.67	0.88	<.001	**1.15**	1.03	1.27	0.011	**1.31**	1.10	1.56	0.002
G2 Maghreb	**0.46**	0.37	0.56	<.001	1.10	0.98	1.23	0.117	0.93	0.77	1.11	0.436
Contrasts												
South-Europe: G2 vs G1	0.96	0.76	1.22	0.743	1.14	0.91	1.41	0.248	**1.66**	1.05	2.64	0.032
Maghreb: G2 vs G1	**1.74**	1.23	2.46	0.002	<u>1.19</u>	0.99	1.42	0.060	**2.62**	1.74	3.95	<.001
G2: South Europe vs Maghreb	**1.68**	1.33	2.13	<.001	1.05	0.90	1.22	0.556	**1.41**	1.11	1.8	0.005

Majority population= neither G1 nor G2.
G1 and G2: first and second generations of immigrants
Adjustments for: generation and region of origin + age (linear and quadratic terms), sex, household characteristics (living in a couple, household size), standard of living and geographic location (living in the Paris metropolitan area or not).
RR risk-ratio, LCL-UCL: lower and upper 95% confidence interval limits.
Reference=majority population (neither G1 nor G2), except in the "Contrasts" panel.
Bold and underlined type: p-value<0.05 and <0.1 respectively.

Supplementary Table 4-1: multivariate modelling of substance use according to language practice adjusting only for age and sex

	Alcohol				Smoking				Cannabis			
	RR	LCL	UCL	P	RR	LCL	UCL	P	RR	LCL	UCL	P
Comparison to the majority population												
G2 South Europe												
HL-speaker	**0.71**	0.58	0.88	0.002	1.11	0.94	1.31	0.191	1.10	0.81	1.45	0.539
Non-HL-speaker	**0.80**	0.68	0.95	0.008	**1.19**	1.04	1.36	0.011	**1.48**	1.19	1.81	<.001
G2 Maghreb												
HL-speaker	**0.18**	0.12	0.28	<.001	0.89	0.75	1.05	0.169	0.80	0.60	1.03	0.096
Non-HL-speaker	**0.71**	0.56	0.88	0.003	**1.40**	1.20	1.62	<.001	1.23	0.96	1.54	0.084
Contrasts												
G2 vs G1 (ref.)												
South-Europe												
HL-speaker	0.87	0.65	1.17	0.359	1.13	0.87	1.45	0.356	1.37	0.82	2.31	0.232
Non-HL-speaker	0.97	0.75	1.26	0.824	1.19	0.94	1.51	0.139	**1.80**	1.11	2.92	0.018
Maghreb												
HL-speaker	0.69	0.41	1.17	0.166	1.07	0.85	1.33	0.579	**2.28**	1.44	3.63	<.001
Non-HL-speaker	**2.58**	1.77	3.74	<.001	**1.58**	1.29	1.94	<.001	**3.27**	2.10	5.10	<.001
G2: Non-HL-speaker vs *HL-speaker* (ref.)												
G2 South-Europe	1.12	0.86	1.47	0.401	1.05	0.86	1.30	0.624	1.31	0.92	1.89	0.138
G2 Maghreb	**3.81**	2.39	6.35	<.001	**1.55**	1.24	1.94	<.001	**1.47**	1.04	2.10	0.030

Majority population= neither G1 nor G2.
Reference=majority population, except in the "Contrasts" panel.
G1 and G2: first and second generations of immigrants.
Adjustment on generation (G1, G2), region of origin and language ("speakers" and "non-speakers") + sex and age (linear and quadratic terms).
HL-speaker / non-HL-speaker: speaker of the Heritage Language at home / non-speaker of the HL i.e. exclusive speaker of French, at age 15
RR risk-ratio, LCL-UCL: lower and upper 95% confidence interval limits.
Bold and underlined type: p-value<0.05 and <0.1 respectively.

Supplementary Table 4-2: Multivariable modelling of substance use according to language practice adjusting only for sex, age, household characteristics, standard of living and geographic location

	**Alcohol**				**Smoking**				**Cannabis**			
	RR	LCL	UCL	P	RR	LCL	UCL	P	RR	LCL	UCL	P
Comparison to the majority population												
G2 South Europe												
HL-speaker	**0.71**	0.57	0.88	0.002	1.13	0.95	1.32	0.160	1.08	0.80	1.43	0.602
Non-HL-speaker	**0.81**	0.68	0.95	0.010	**1.16**	1.01	1.33	0.030	**1.47**	1.19	1.81	<.001
G2 Maghreb												
HL-speaker	**0.19**	0.12	0.29	<.001	0.89	0.75	1.05	0.180	<u>0.79</u>	0.60	1.02	0.085
Non-HL-speaker	**0.73**	0.58	0.91	0.008	**1.33**	1.14	1.53	<.001	1.08	0.84	1.35	0.542
Contrasts												
G2 vs G1 (ref.)												
South-Europe												
HL-speaker	0.88	0.65	1.19	0.408	1.08	0.84	1.40	0.550	1.34	0.80	2.31	0.280
Non-HL-speaker	0.98	0.76	1.29	0.901	1.09	0.86	1.39	0.474	**1.72**	1.07	2.88	0.031
Maghreb												
HL-speaker	0.78	0.45	1.31	0.354	1.05	0.83	1.32	0.681	**2.36**	1.48	3.84	<.001
Non-HL-speaker	**2.56**	1.76	3.76	<.001	**1.46**	1.19	1.80	<.001	**3.00**	1.94	4.76	<.001
G2: Non-HL-speaker vs *HL-speaker* (ref.)												
G2 South-Europe	1.07	0.82	1.41	0.614	1.01	0.82	1.25	0.907	1.29	0.90	1.86	0.177
G2 Maghreb	**3.49**	2.17	5.85	<.001	**1.45**	1.16	1.82	0.001	<u>1.36</u>	0.95	1.96	0.091

Majority population= neither G1 nor G2.
G1 and G2: first and second generations of immigrants
Adjustment on generation, region of origin and language (“speakers” and “non-speakers”) + age (linear and quadratic terms), sex, household characteristics (living in a couple, household size), standard of living, and geographic location (living in the Paris metropolitan area).
HL-speaker / non-HL-speaker: speaker of the Parental Foreign Language at home / non-speaker of the HL i.e. exclusive speaker of French, at age 15

RR risk-ratio, LCL-UCL: lower and upper 95% confidence interval limits.
Reference=majority population (neither G1 nor G2), except in the “Contrasts” panel.
Bold and underlined type: p-value<0.05 and <0.1 respectively.

Supplementary Table 5: Multivariable modelling of substance use according to parentage

	Alcohol				Smoking				Cannabis			
**Model 3**	**RR**	**LCL**	**UCL**	**P**	**RR**	**LCL**	**UCL**	**P**	**RR**	**LCL**	**UCL**	**P**
Comparison to the majority population												
G2 South Europe												
Homogenous	**0.70**	0.55	0.89	0.003	0.97	0.8	1.16	0.717	1.02	0.71	1.47	0.896
Mixed	**0.84**	0.72	0.98	0.028	**1.17**	1.03	1.32	0.017	**1.41**	1.15	1.7	<0.001
G2 Maghreb												
Homogenous	**0.27**	0.19	0.37	<0.001	0.89	0.76	1.04	0.134	**0.67**	0.50	0.89	0.005
Mixed	0.80	0.62	1.02	0.078	**1.21**	1.03	1.41	0.021	**1.28**	1.02	1.61	0.036
Contrasts												
G2 South Europe:												
Mixed vs homogenous	1.20	0.90	1.58	0.211	1.21	0.97	1.51	0.098	1.37	0.91	2.06	0.128
G2 Maghreb:												
Mixed vs homogenous	**2.97**	1.95	4.53	<.001	**1.36**	1.09	1.7	0.006	**1.91**	1.34	2.73	<.001

Majority population= neither G1 nor G2.
Reference=majority population, except in the “Contrasts” panel.
G1 and G2: first and second generations of immigrants
Adjustment on generation (G1, G2), migratory region of origin and parentage (“homogenous” and “mixed”) + age (linear and quadratic terms), educational level (ridit score), household characteristics (living in a couple, household size), socioeconomic status (perceived financial situation, standard of living, deprivation index of the city of residence), and geographic location (living in the Paris metropolitan area).
Homogenous: both parents originating from the world region of immigration; Mixed: one parent originating from the world region of immigration, the other from France.
RR risk-ratio, LCL-UCL: lower and upper 95% confidence interval limits.
Reference=majority population, except in the “Contrasts” panel.
Bold and underlined type: p-value<0.05 and <0.1 respectively.

Supplementary Table 5-1: Multivariable modelling of substance use according to parentage adjusting only for sex and age

	Alcohol				Smoking				Cannabis			
**Model 3**	**RR**	**LCL**	**UCL**	**P**	**RR**	**LCL**	**UCL**	**P**	**RR**	**LCL**	**UCL**	**P**
Comparison to the majority population												
G2 South Europe Homogenous	**0.68**	0.53	0.85	0.001	1.02	0.84	1.22	0.869	1.06	0.72	1.49	0.770
Mixed	**0.81**	0.69	0.95	0.009	**1.23**	1.09	1.39	<.001	**1.42**	1.16	1.72	<.001
G2 Maghreb Homogenous	**0.24**	0.17	0.34	<.001	0.94	0.80	1.10	0.470	**0.69**	0.52	0.90	0.010
Mixed	**0.73**	0.56	0.92	0.011	**1.40**	1.19	1.64	<.001	**1.39**	1.10	1.74	0.004
Contrasts												
G2 South Europe: Mixed vs homogenous	1.20	0.91	1.59	0.199	<u>1.21</u>	0.97	1.52	0.086	1.35	0.90	2.02	0.151
G2 Maghreb: Mixed vs homogenous	**2.98**	1.95	4.54	<.001	**1.49**	1.19	1.85	<.001	**2.02**	1.42	2.87	<.001

Majority population= neither G1 nor G2.
Reference=majority population, except in the “Contrasts” panel.
G1 and G2: first and second generations of immigrants
Adjustment on generation (G1, G2), migratory region of origin and parentage (“homogenous” and “mixed”) + age (linear and quadratic terms).
Homogenous: both parents originating from the world region of immigration; Mixed: one parent originating from the world region of immigration, the other from France.
RR risk-ratio, LCL-UCL: lower and upper 95% confidence interval limits.
Reference=majority population, except in the “Contrasts” panel.
Bold and underlined type: p-value<0.05 and <0.1 respectively.

Supplementary Table 5-2: Multivariable modelling of substance use according to parentage adjusting only for sex, age, household characteristics, standard of living and geographic location

	Alcohol				Smoking				Cannabis			
**Model 3**	**RR**	**LCL**	**UCL**	**P**	**RR**	**LCL**	**UCL**	**P**	**RR**	**LCL**	**UCL**	**P**
Comparison to the majority population												
G2 South Europe												
Homogenous	**0.68**	0.53	0.85	0.001	1.01	0.83	1.21	0.934	1.02	0.69	1.44	0.916
Mixed	**0.82**	0.70	0.95	0.011	**1.22**	1.07	1.38	0.002	**1.42**	1.17	1.72	<.001
G2 Maghreb												
Homogenous	**0.26**	0.18	0.35	<.001	0.95	0.80	1.10	0.481	**0.66**	0.49	0.87	0.004
Mixed	**0.76**	0.59	0.96	0.029	**1.31**	1.12	1.54	<.001	**1.26**	0.99	1.57	0.050
Contrasts												
G2 South Europe:												
Mixed vs homogenous	1.21	0.91	1.60	0.185	1.21	0.97	1.51	0.095	1.40	0.93	2.10	0.108
G2 Maghreb:												
Mixed vs homogenous	**2.98**	1.95	4.54	<.001	**1.39**	1.12	1.73	0.003	**1.90**	1.33	2.71	<.001

Majority population= neither G1 nor G2.
Reference=majority population, except in the "Contrasts" panel.
G1 and G2: first and second generations of immigrants
Adjustment on generation (G1, G2), migratory region of origin and parentage ("homogenous" and "mixed") + age (linear and quadratic terms), household characteristics (living in a couple, household size), standard of living and geographic location (living in the Paris metropolitan area).
Homogenous: both parents originating from the world region of immigration; Mixed: one parent originating from the world region of immigration, the other from France.
RR risk-ratio, LCL-UCL: lower and upper 95% confidence interval limits.
Reference=majority population, except in the "Contrasts" panel.
Bold and underlined type: p-value<0.05 and <0.1 respectively.
